# Preparation and performance of crosslinked poly(arylene ether)s containing azobenzene chromophores

**DOI:** 10.1080/15685551.2017.1371823

**Published:** 2017-09-13

**Authors:** Yuxuan Zhang, Shuang Wang, Zhonghui Wang, Saisai Gai, Xi-Ming Song

**Affiliations:** ^a^ Liaoning Key Laboratory for Green Synthesis and Preparative Chemistry of Advanced Materials, College of Chemistry, Liaoning University, Shenyang, China

**Keywords:** Poly(arylene ether)s, azobenzene, photoinduced birefringence, crosslink

## Abstract

A series of novel poly(arylene ether)s with crosslinked groups and different azobenzene chromophores contents (azo-CPAEs: PAE-allyl20%-azo20%, PAE-allyl20%-azo40%, PAE-allyl20%-azo60%) were synthesized from a new bisfluoro monomer, (2,6-difluorophenyl)-(4-hydroxyphenyl)methanone. Their chemical structures were characterized by means of UV-vis and FI-IR. The thermal properties of the polymers were investigated by TGA and DSC, indicating the polymers had high glass transition temperatures (T_g_ > 147 °C) and good thermal stability (T_d5_ > 360 °C) even when the contents of azobenzene chromophores was high to 60%. And the influence of thermal crosslinking on the performance of PAE-allyl20%-azo20%, a typical one of the series, was investigated. T_g_ of PAE-allyl20%-azo20% increased with the increase of heating time when heat-treated at 250 °C for 20, 40 and 60 min, indicating the crosslink degree of the polymer increased. After heat-treated for 60 min, T_g_ of PAE-allyl20%-azo20% increased to 175 °C from 147 °C before thermal crosslinking. Upon irradiation with a 532 nm neodymium doped yttrium aluminum garnet (Nd:YAG) laser beam, the remnant value of the polymer PAE-allyl20%-azo20% before and after the thermal crosslinking were 81 and 96%, respectively, meaning that the PAE-allyl20%-azo20% after thermal crosslink showed more stable photoinduced alignment than that before thermal crosslinking.

## 1. Introduction

Azobenzene-containing polymers (azo-polymers) have attracted much attention because of their vast application in optical data storage, optical switches and nonlinear optical materials [1–6]. Due to the unique reversible photoisomerization and photoinduced anisotropy of the azobenzene chromophores, azo-polymers can show a variety of photoresponsive variations, such as photoinduced phase transition, photoinduced surface-relief-gratings, photoinduced bending and photoinduced birefringence [7–11]. Photoinduced birefringence is an important feature of azo-polymers that makes them suitable for optical storage applications. When an azo-polymer film was illuminated by a linearly polarized laser light, the azobenzene chromophores undergo *trans*-*cis*-*trans* photoisomerization accompanied by molecular reorientation. The photoinduced orientation of the azobenzene chromophores was perpendicular to the polarization direction. This molecular reorientation resulted in birefringence property changing in the azobenzene-containing materials, which could be erased by randomization of the alignment with circularly polarized light or heat [11].

For optical storage devices, long-term stability of the optical properties is an important pre-requisite. In recent years, azobenzene chromophores have been introduced into the polymers with high glass transition temperatures because it is a helpful strategy to improve the stability of optical storage [12,13]. Poly(arylene ether)s are a family of high-performance engineering thermoplastics known for their excellent thermal stability. Functionalized poly(arylene ether)s have received considerable attention due to their potential applications in nonlinear optical materials, fluorescent material and proton-exchange membranes [14–16]. In our previous work, we have reported some azobenzene-containing poly(arylene ether)s and investigated their optical properties [17–20]. However, when the excitation light was turned off, photoinduced birefringence of the polymers always exhibit a little decay, which affects the stability of optical storage. The reason was attributed to the thermal reorientation of some azobenzene groups. It has been reported that crosslinking is an effective method to improve the thermal stability of the chromophore’s orientation [21]. Thus, in this work, we designed and synthesized a series of photoresponsive azo-poly(arylene ether)s with azobenzene chromophores and crosslinked groups by direct copolymerization. Their structures were characterized, and their thermodynamic behavior and photoactive behavior were investigated.

## 2. Experimental

### 2.1. Materials

4-((4-Cyanophenyl)diazenyl)phenyl-2,6-difluorobenzoate (DF-AZO-CN) was synthesized according to the literature [22]. Hydroiodic acid, allyl bromide, aluminum chloride and bisphenol A were purchased from Aladdin Chemistry Co. Ltd. 2,6-Difluorobenzoyl chloride was purchased from Ruiding Chemicals Co. Ltd. Anisole and 4,4′-difluorobenzophenone (DFBP) was purchased from TCI. All the solvents were purchased from commercial sources. Potassium carbonate was dried at 120 °C for 24 h.

### 2.2. Measurements

FI-IR spectra (KBr pellet) were recorded on a Nicolet Impact 410 FT-IR spectrophotometer. ^1^H NMR spectra were recorded on a Bruker 400 MHz instrument using dimethysulfoxide-d_6_ (DMSO-d_6_) as solvent, and the chemical shifts (δ) were given in ppm using tetramethylsilane (TMS) as internal reference. UV-visible absorption spectra were recorded on a Perkin-Elmer Lambda 35 UV-vis spectrophotometer at room temperature. Glass transition temperatures (T_g_s) were determined by a Model Mettler DSC instrument with a heating rate of 20 °C/min and under a nitrogen flow of 200 mL/min. Thermo-gravimetric analysis was performed on a Perkin Elmer Pyris 1 TGA analyzer under nitrogen atmosphere at a heating rate of 10 °C/min.

### 2.3. Synthesis

#### 2.3.1. Synthesis of (2,6-difluorophenyl)-(4-methoxyphenyl)methanone (monomer 1, scheme 1)

Under N_2_ atmosphere, a solution of 2,6-difluorobenzoyl chloride (17.655 g, 0.1 mol) in 40 mL CH_2_Cl_2_ was added dropwise into a mixture of anisole (10.813 g, 0.1 mol) and aluminum chloride (AlCl_3_, 14.669 g, 0.11 mol) in 80 mL CH_2_Cl_2_. After stirring for 24 h, the reaction mixture was filtered. After evaporating the filtrate, the resulting precipitate was washed by deionized water and dried. The crude product was recrystallized from toluene, and the monomer 1 was obtained as white crystal. (yield: 81%). IR (KBr, cm^−1^): 2938 (–OCH_3_); ^1^H NMR (DMSO-d_6_, δ, ppm): 7.81 (d, 2H), 7.64–7.72 (m, 1H), 7.31 (t, 2H), 7.13 (d, 2H), 3.88 (s, 3H, –OCH_3_).

#### 2.3.2. Synthesis of (2,6-difluorophenyl)-(4-hydroxyphenyl)methanone (monomer 2, scheme 1)

The monomer 1 (12.4115 g, 0.05 mol) was dissolved in a mixture of 60 mL acetic acid and 30 mL hydroiodic acid, and refluxed for 9 h. After being poured into deionized water, the precipitate was collected by filtration. The crude product was recrystallized from toluene, and the monomer 2 was obtained as white crystal. (yield: 92%). IR (KBr, cm^−1^): 3355 (–OH); ^1^H NMR (DMSO-d_6_, δ, ppm): 10.79 (s, 1H, –OH), 7.69(d, 2H), 7.62–7.66 (m, 1H), 7.28(t, 2H), 6.92 (d, 2H).

#### 2.3.3. Synthesis of (4-(allyloxy)phenyl)-(2,6-difluorophenyl)methanone (monomer 3, scheme 1)

The monomer 2 (2.1926 g, 0.04 mol), allyl bromide (6.049, 0.05 mol), sodium hydroxide (2.0 g, 0.05 mol) and 20 mL deionized water was heated under reflux with stirring for 48 h. The reaction mixture was poured into aqueous sodium hydroxide (800 ml, 0.2 mol) under stirring, and then the precipitate was collected by filtration and dried. (yield: 62%). IR (KBr, cm^−1^): 1260 (–Ph–O–C–); ^1^H NMR (DMSO-d_6_, δ, ppm): 7.78 (d, 2H), 7.64–7.71 (m, 1H), 7.14 (d, 2H), 6.01–6.11 (m, 1H), 5.43 (d, 1H), 5.31(d, 1H), 4.71(d, 2H).

#### 2.3.4. Synthesis of the polymers (scheme 2)

The synthetic procedure of azo-CPAE copolymers was shown as follows. For PAE-allyl20%-azo20%, monomer 3 (0.5205 g, 0.002 mol), DF-AZO-CN (0.7266 g, 0.002 mol), DFBP (1.3092 g, 0.006 mol), bisphenol A (2.2829 g, 0.01 mol), K_2_CO_3_ (1.5201 g, 0.011 mol), DMAC (17 ml) and toluene (10 ml) were put into a three-necked flask and the flask was purged with nitrogen. The reaction mixture was refluxed at 130 °C for 3 h to ensure complete dehydration. After dehydration and removal of toluene, the reaction mixture was heated at 150–160 °C for 8 h under nitrogen atmosphere. After being poured into deionized water, the precipitate was collected by filtration. The crude product was washed with hot deionized water and ethanol several times in sequence. The resulting product was dried at 80 °C under vacuum for 24 h and PAE-allyl20%-azo20% was obtained as orange powder. The yield was 91%. The copolymers were prepared by varying the mole fractions of monomer 3 (n), DF-AZO-CN (m) and BHPVA (l). Copolymers with differing n/m/l ratios 2:2:6, 2:4:4 and 2:6:2 were prepared and designated as PAE-allyl20%-azo20%, PAE-allyl20%-azo40% and PAE-allyl20%-azo60%, respectively. In addition, two other polymers with differing n/m/l ratios 2:0:8 and 0:2:8 were also prepared and designated as PAE-allyl20% and PAE-azo20%, respectively.

### 2.4. Preparation of film samples

The polymers were dissolved in cyclohexanone (10 wt%) and filtered through 0.45 μm syringe filter membranes. The polymer films were obtained by casting the solution of polymers onto glass substrates. The thickness of the films was about 10 μm for the photoinduced birefringence experiment. The film samples were dried under vacuum at 120 °C for 24 h.

## 3. Results and discussion

### 3.1. Synthesis and characterization

**Figure 1.**
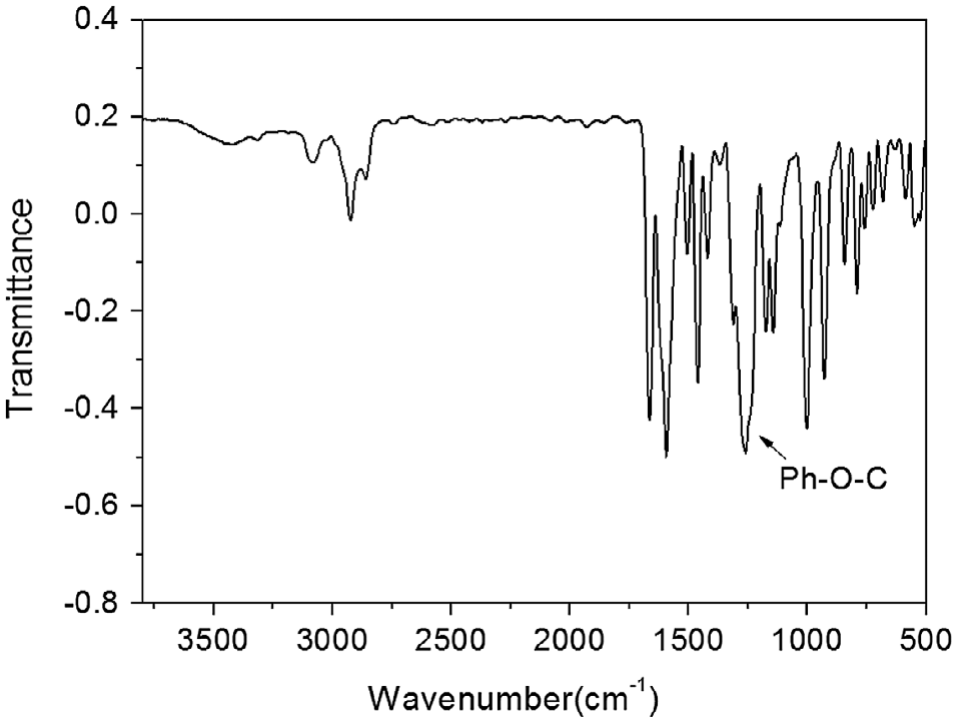
IR (KBr) spectrum of monomer 3.

**Figure 2.**
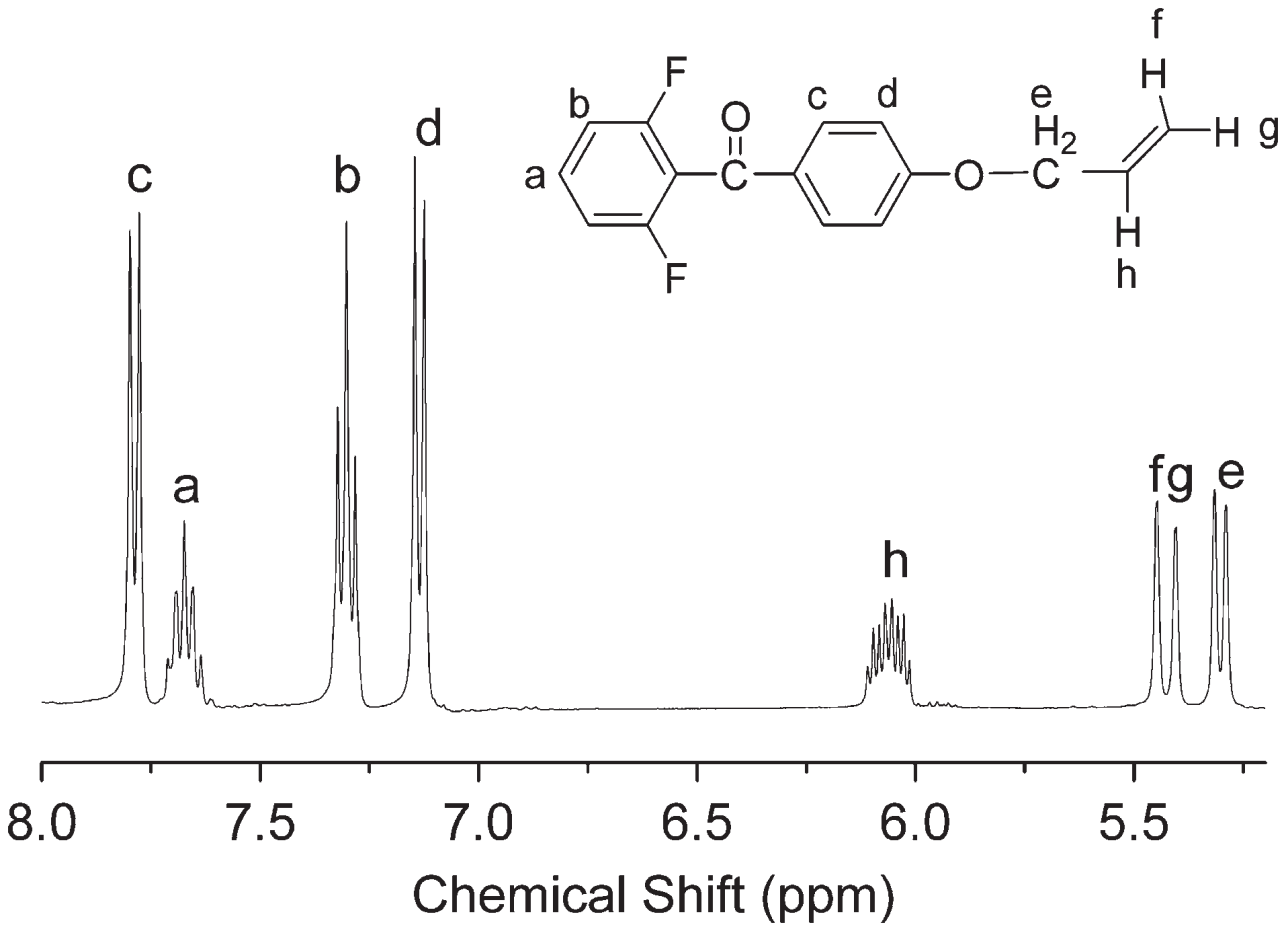
^1^H NMR spectrum of monomer 3 in DMSO-d_6_.

**Figure 3.**
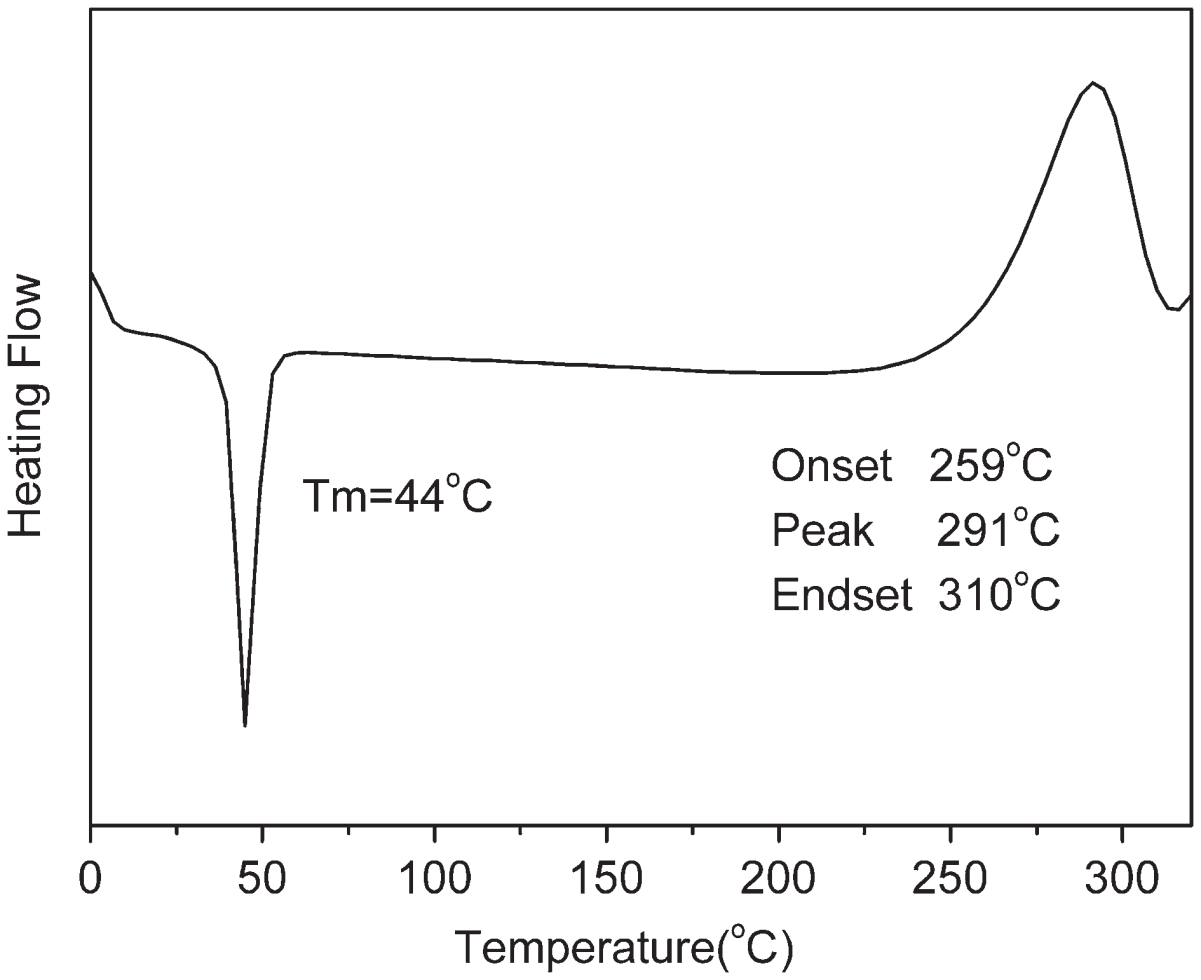
DSC curve of monomer 3.

**Scheme 1.**
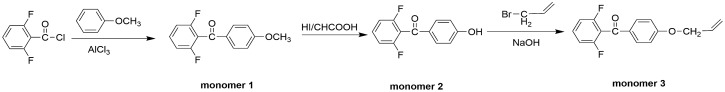
Synthetic route of monomer 3.

We designed a new bisfluoro monomer (monomer 3) containing crosslinked group and the reaction mechanism for the preparation of the monomer is illustrated in Scheme 1. As shown in Figures 1 and 2, the structure of monomer 3 was confirmed by IR and ^1^H NMR. The IR spectrum showed the characteristic band of –Ph–O–C– stretching vibration at 1260 cm^−1^, respectively. In the ^1^HNMR spectrum of monomer 3, all of the signals are well agreement with the expected structure. Figure 3 showed the DSC curve of monomer 3, it could be observed that an exothermic peak at 291 °C corresponding to the crosslinked group of monomer 3.

**Figure 4.**
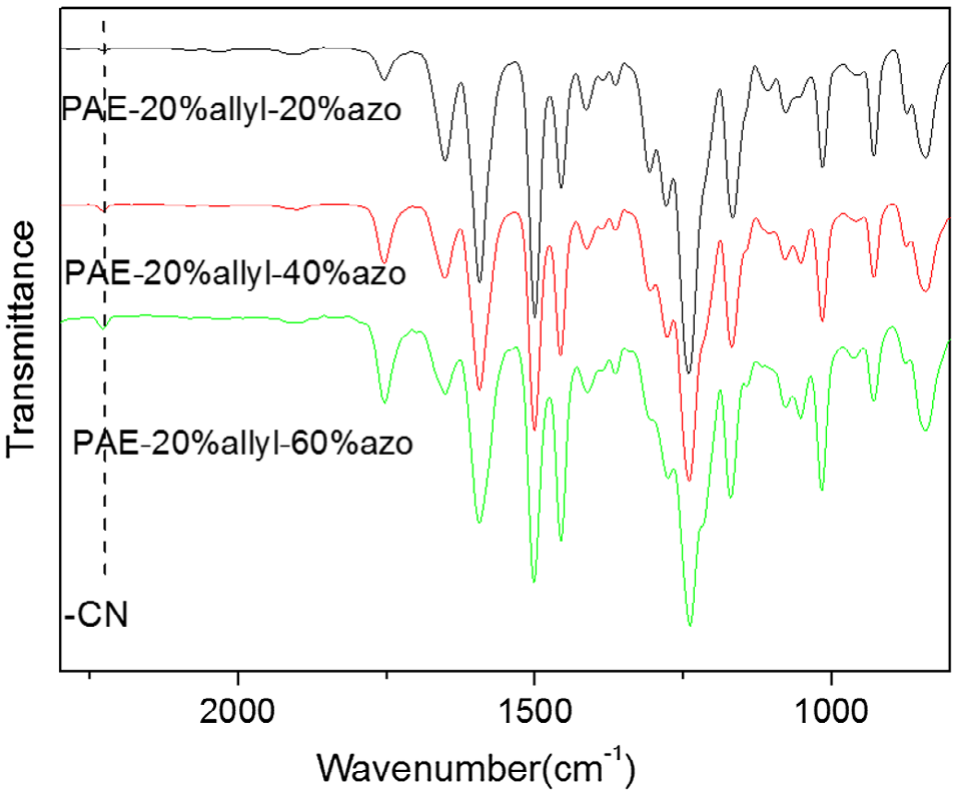
IR (KBr) spectra of azo-CPAE copolymers.

**Figure 5.**
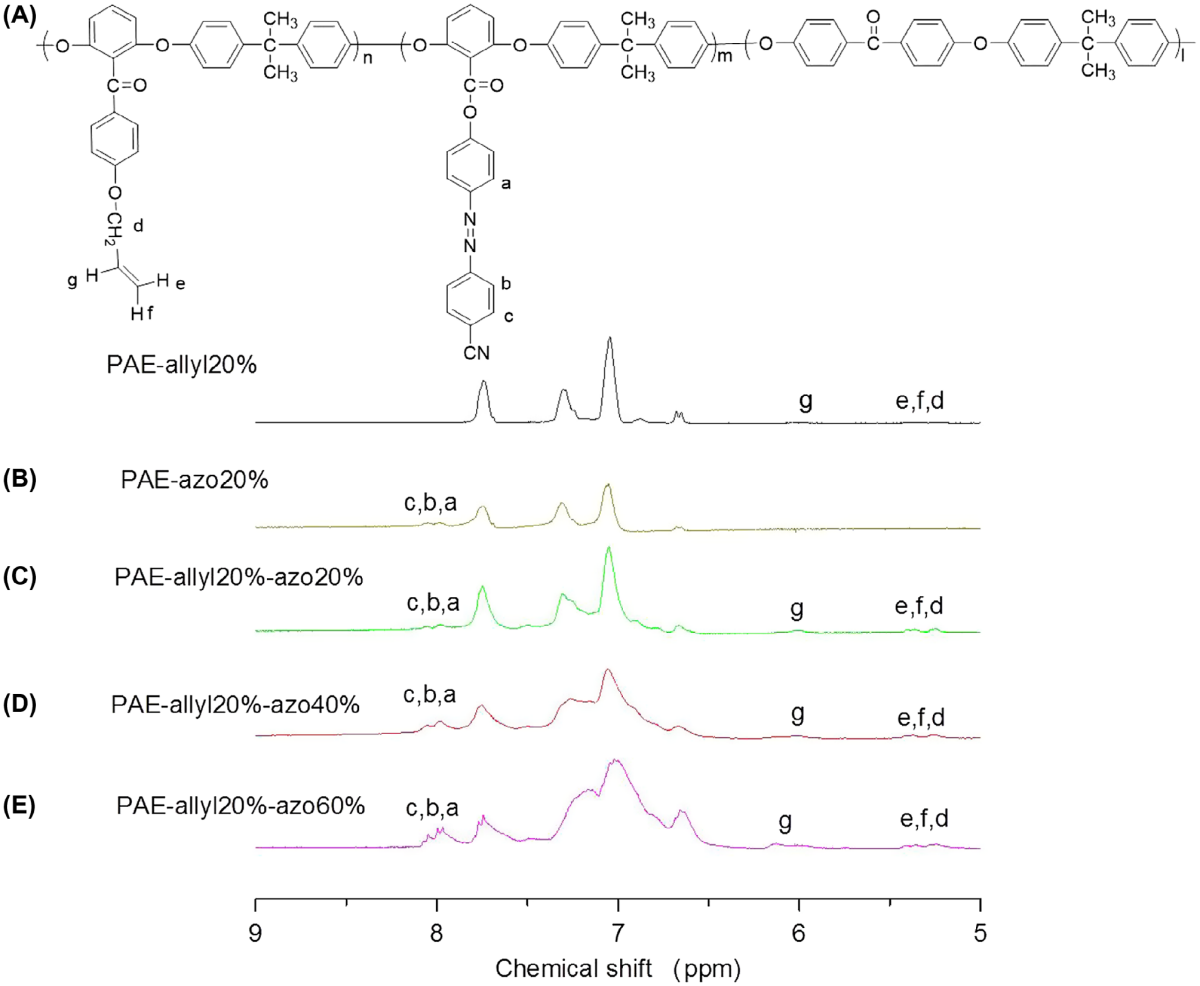
^1^H NMR spectra of the polymers:(A) PAE-allyl20%; (B) PAE-azo20%; (C) PAE-allyl20%-azo20; (D)PAE-allyl20%-azo40%; (E) PAE-allyl20%-azo60%.

**Figure 6.**
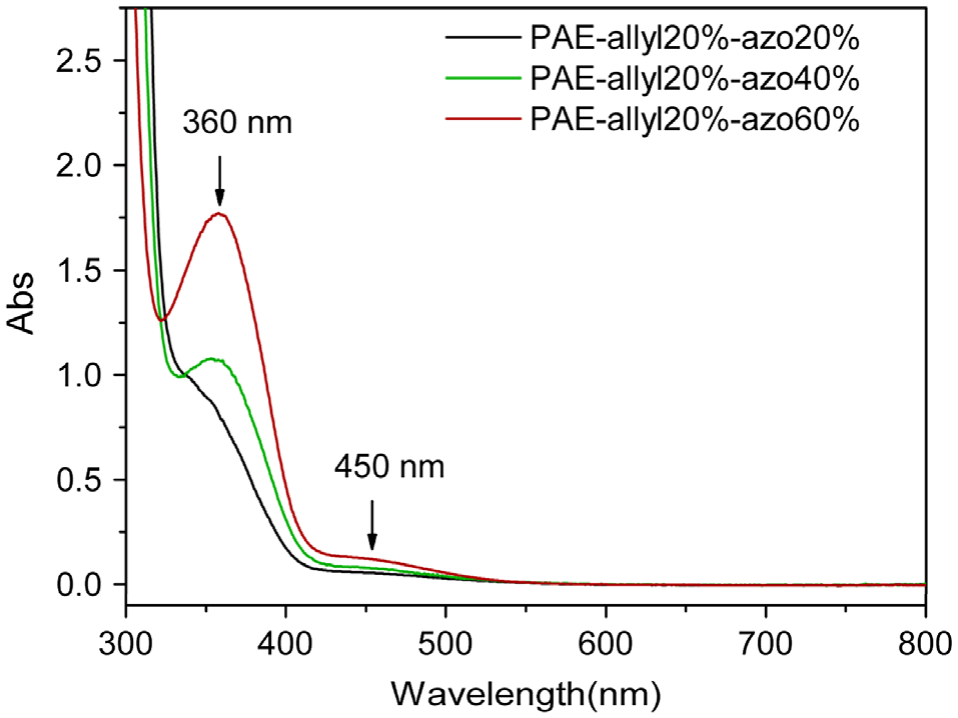
UV-vis spectra of azo-CPAE copolymers in DMF solution at 25 °C.

**Figure 7.**
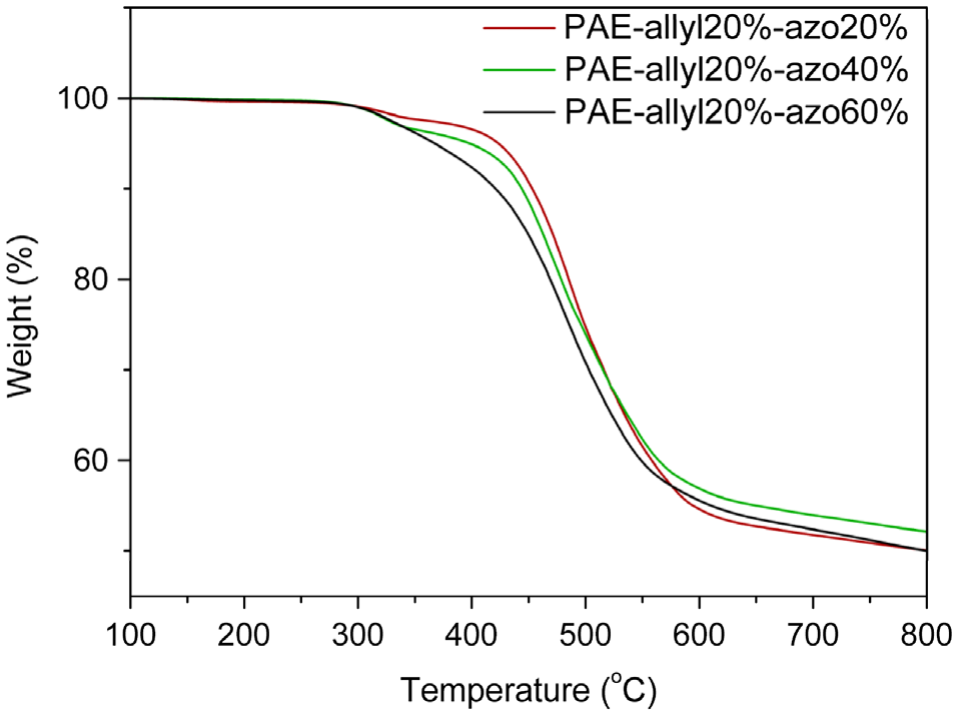
TGA curve of azo-CPAE copolymers in nitrogen.

**Figure 8.**
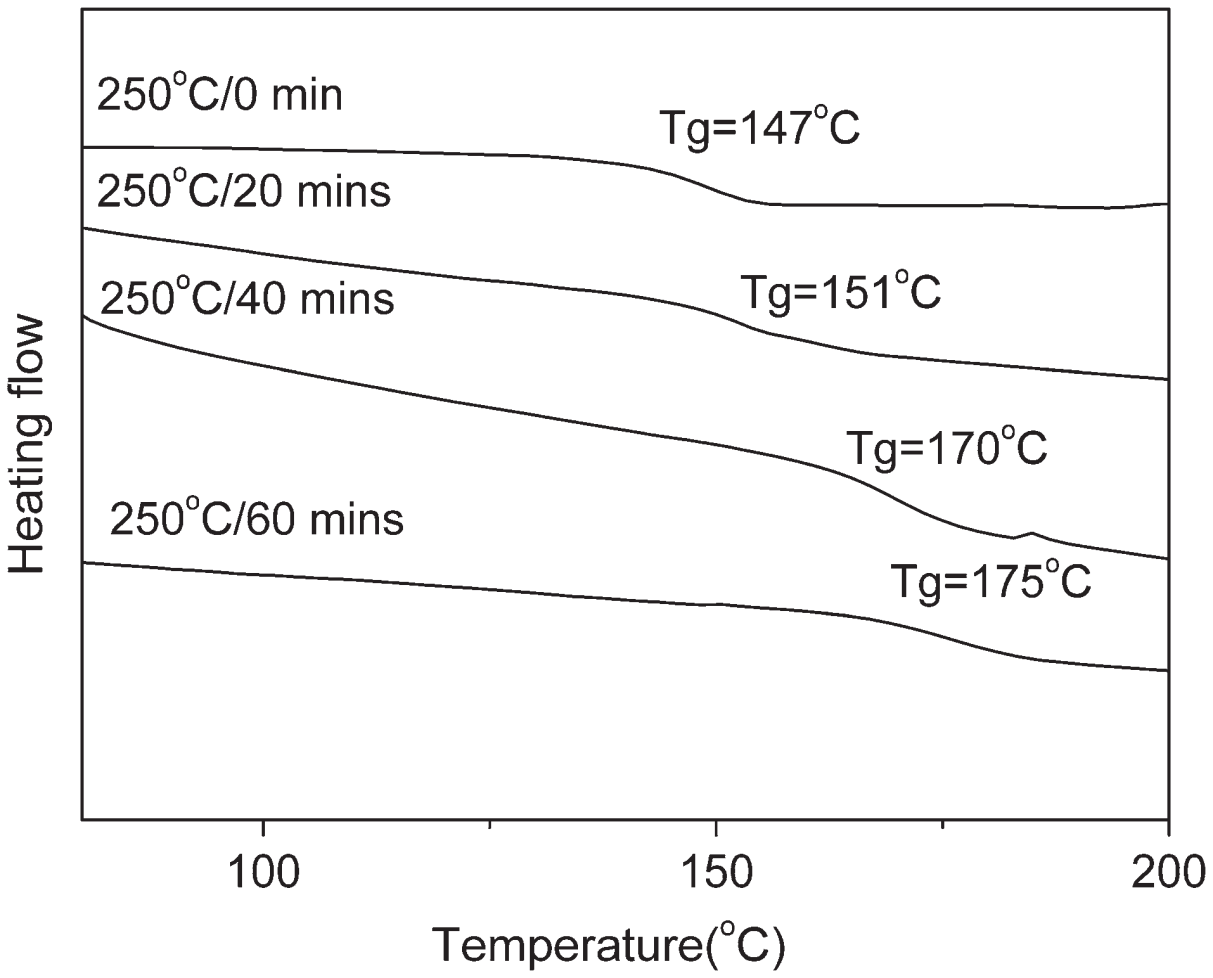
DSC curves of PAE-allyl20%-azo20% heated at 250 °C for different time: 0, 20, 40 and 60 min.

**Figure 9.**
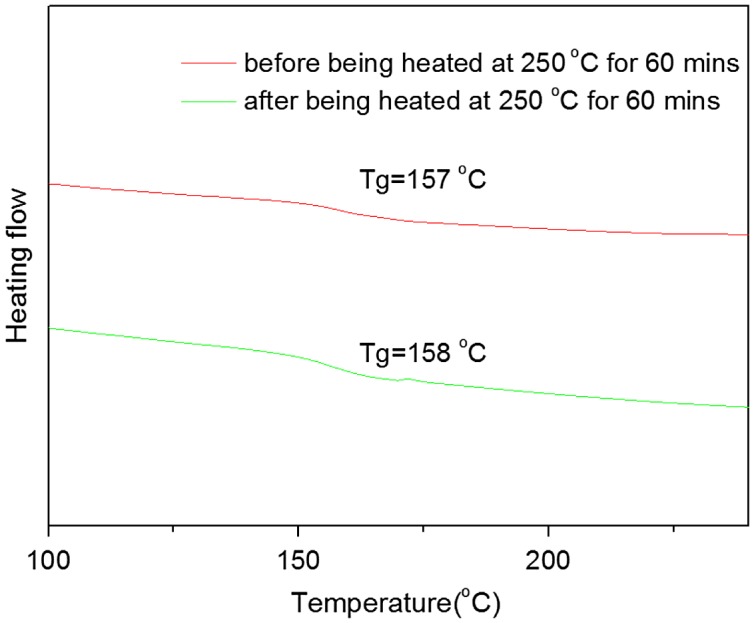
DSC curves of PAE-azo20% before and after being heated at 250 °C for 60 min.

**Figure 10.**
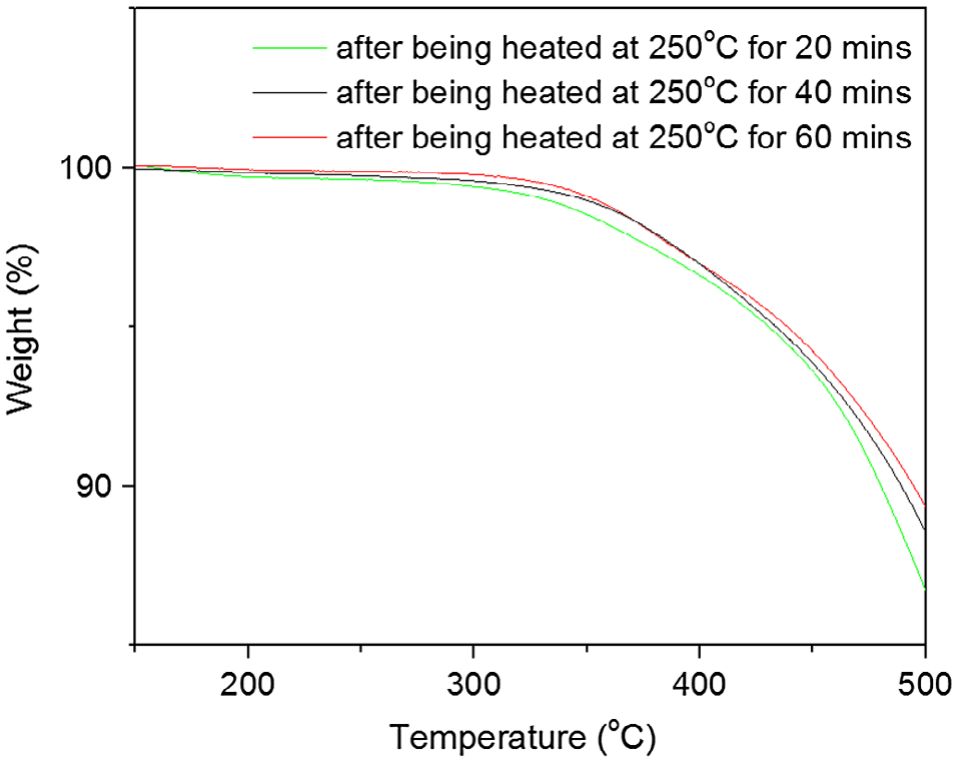
TGA curves of PAE-allyl20%-azo20% after being heated at 250 °C for different time (20, 40 and 60 min).

**Figure 11.**
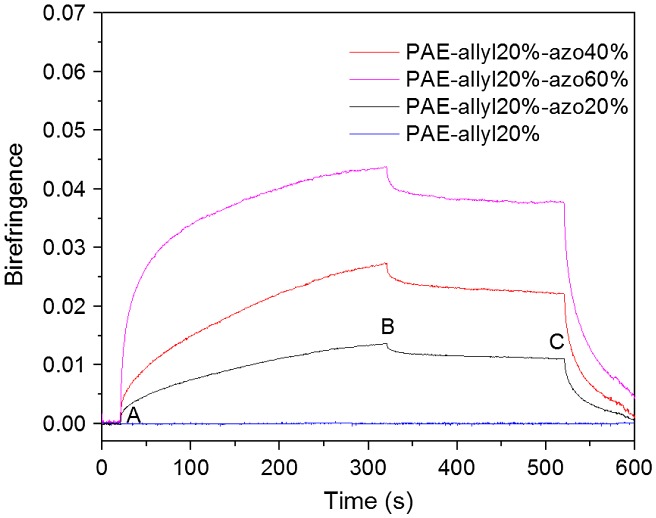
Typical behaviour of the photoinduced birefringence of PAE-allyl20%, PAE-allyl20%-azo20%, PAE-allyl20%-azo40% and PAE-allyl20%-azo60%: at point A, the writing laser was turned on; at point B, the writing laser was turned off; at point C, the circularly polarized light was turned on.

**Figure 12.**
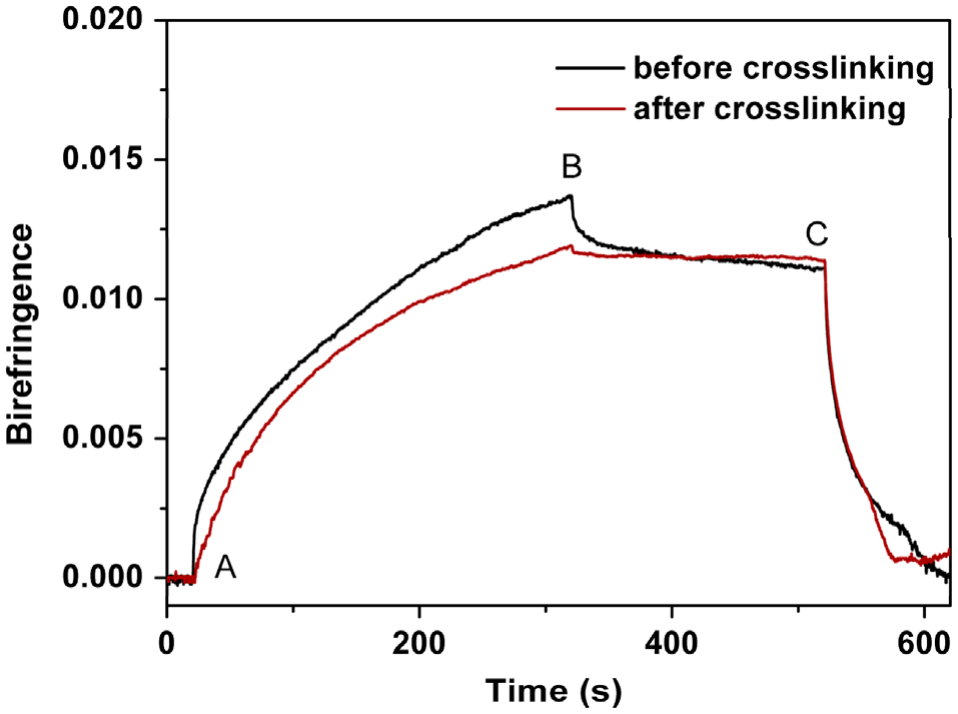
Typical behaviour of the photoinduced birefringence of PAE-allyl20%-azo20% before and after crosslinking.

**Figure 13.**
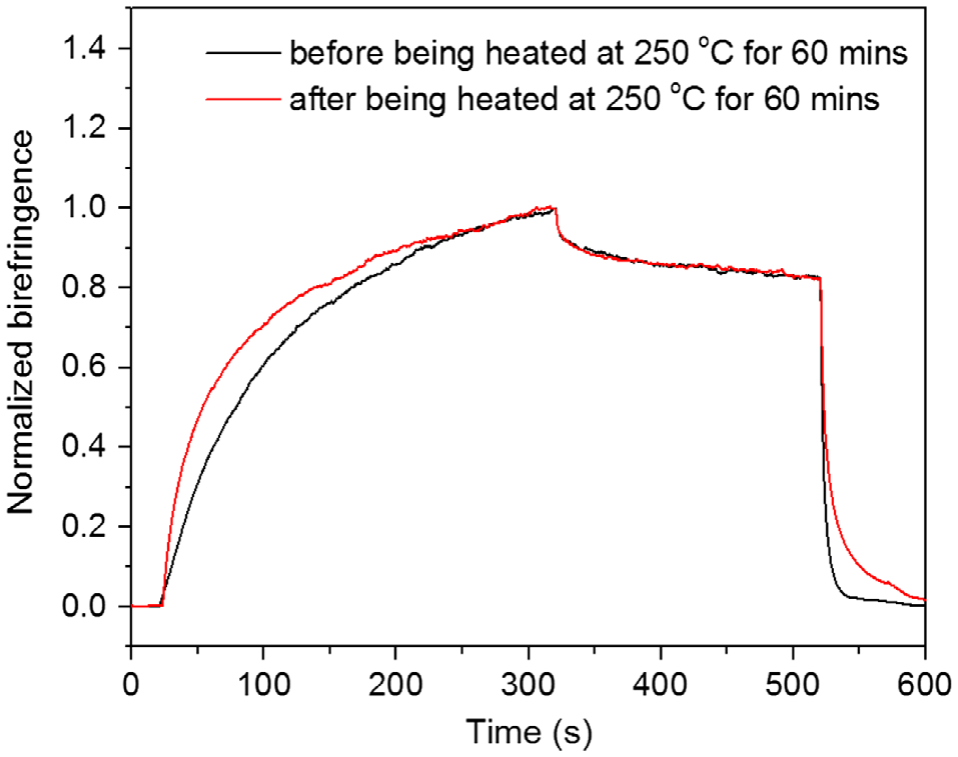
Normalized photoinduced birefringence of PAE-azo20% before and after being heated at 250 °C for 60 min.

**Scheme 2.**
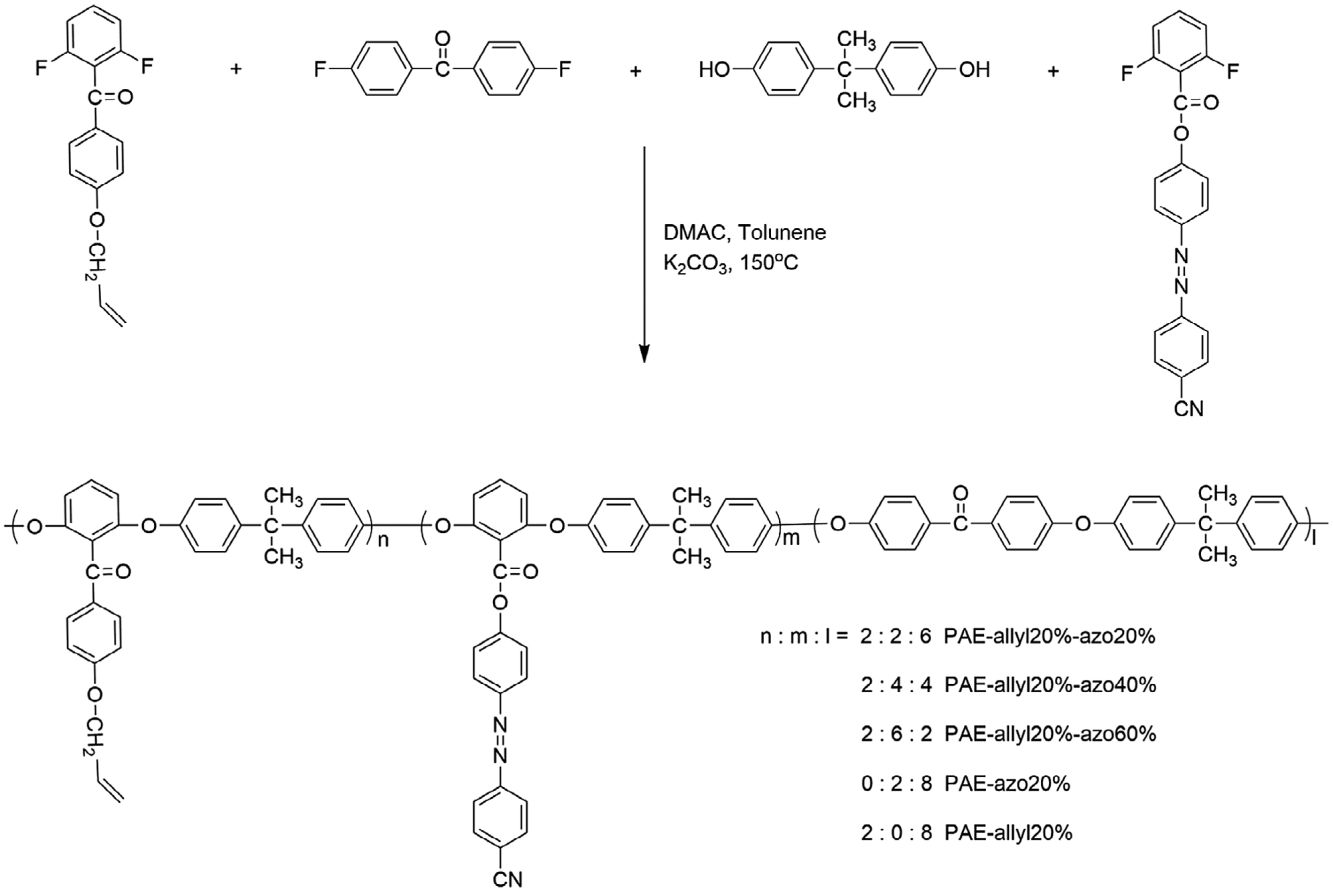
Synthesis routes to the polymers.

Poly(arylene ether)s containing azobenzene chromophores and crosslinked groups were synthesized by a typical nucleophilic substitution polycondensation reaction, as shown in Scheme 2. From Table 1, it could be seen that all the azo-CPAE copolymers had number average molecular weights above 7 × 10^3^ g/mol. All of the azo-CPAE copolymers showed good solubility in common organic solvents such as tetrahydrofuran, cyclohexanone, dimethylacetamide, *N,N*-dimethylformamide and *N*-methyl-2-pyrrolidone.

**Table 1.** Properties of azo-CPAE copolymers.

Polymer	M_n_	M_w_/M_n_	T_g_ (°C)^a^	T_d5_ (°C)^b^	T_d10_ (°C)^c^
PAE-allyl20%-azo20%	9.7 × 10^3^	2.1	147	423	453
PAE-allyl20%-azo40%	8.5 × 10^3^	1.8	148	397	433
PAE-allyl20%-azo60%	7.1 × 10^3^	2.2	151	366	420

^a^ Glass transition temperature by DSC.

^b^ 5% weight-loss temperatures were detected at a heating rate of 10 °C/min in nitrogen with a gas flow of 100 mL/min.

^c^ 10% weight-loss temperatures were detected at a heating rate of 10 °C/min in nitrogen with a gas flow of 100 mL/min.

The chemical structures of azo-CPAE copolymers were confirmed by IR and UV-vis spectra. The IR spectra of azo-CPAE copolymers were shown in Figure 4. The IR spectra of azo-PAE copolymers showed characteristic absorption band of –CN groups at 2230 cm^−1^. Figure 5 showed the typical ^1^H NMR spectra of the polymers in DMSO-d_6_ with signal assignment. As shown in Figure 5, the peaks at around 1.60 ppm could be assigned to the signals of the protons of –CH_3_ group. The peaks at 5.27, 5.38 and 6.03 ppm in Figure 5(A), (C), (D) and (E) could be assigned to the signals of the protons d, e and f by comparing the ^1^H NMR spectrum of PAE-azo20% (Figure 5(B)) with the others in Figure 5. The peaks at around 7.99 and 8.05 ppm in the Figure 5(B), (C), (D) and (E) could be assigned to the signals of protons c, b and a by comparing the ^1^H NMR spectrum of PAE-allyl20% (Figure 5(A)) with the others in Figure 5. On the basis of ^1^H NMR spectra, the contents of azobenzene chromophores in azo-CPAEs were calculated to be 16, 33 and 47%, respectively, from the integral ratio of the protons of the a, b and c. The UV-vis spectra of azo-CPAE copolymers in DMF solution were shown in Figure 6. As shown in Figure 6, the characteristic absorption bands at around 360 and 450 nm could be observed which corresponded to *π*–π* and *n*–π* transitions resulted from the intramolecular charge transfer of the azobenzene chromophores, respectively [23]. The absorption intensity of azo-CPAE copolymers at 360 nm increased with the contents of azobenzene chromophores.

### 3.2. Thermal properties of azo-CPAE copolymers

The detailed experimental data from the DSC analysis were illustrated in Table 1. Due to the aromatic structure, all the azo-CPAE copolymers showed high T_g_s. Figure 6 showed the TGA analysis curves of polymers, and the detailed experimental data from the TGA analysis were illustrated in Table 1. From Table 1, it could be observed that the temperatures at 5% weight loss (T_d5_) of azo-CPAE copolymers were decreased with the contents of azobenzene chromophores. But even the contents of azobenzene chromophores were high to 60%, the T_d5_ of all the polymers were above 360 °C, indicating their good thermal stability (Figure 7).

### 3.3. Crosslink behaviors of azo-CPAE copolymers

The influence of thermal crosslinking on the performance of PAE-allyl20%-azo20%, a typical one of the series, was investigated by DSC test. Figure 8 showed the DSC curves of PAE-allyl20%-azo20% was heated at 250 °C for different time (0, 20, 40, 60 min). It could be observed that the T_g_ value of PAE-allyl20%-azo20% increase from 147 °C to 175 °C with the heating time increased from 0 to 60 min at 250 °C, indicating the crosslink degree of the polymer increased.

Figure 9 showed the DSC curves of PAE-azo20% before and after being heated at 250 °C for 60 min. It could be seen that there is no change of T_g_ after being heated at 250 °C for 60 min, and the results further confirmed the changes of the T_g_ of PAE-allyl20%-azo20% after being heated at 250 °C for different time were caused by the crosslinking of allyl groups.

Figure 10 showed the TGA curves of PAE-allyl20%-azo20% after being heated at 250 °C for different time (20, 40 and 60 min). It could be seen the temperatures at 5% weight loss (T_d5_) of PAE-allyl20%-azo20% were increased with the heating time.

### 3.4. Photoinduced birefringence of photochromic film samples

The experimental setup for photoinduced birefringence experiment has been described by our group previously [17], and only some details are given here. The photoinduced birefringence was evaluated by a pump-probe optical system, which was made up of writing processes (linearly polarized pump on), relaxation processes (pump off), and erasing processes (circularly polarized pump on). The photoinduced birefringence was induced by irradiation of the film samples with a linearly polarized laser at 532 nm with 63 mW/cm^2^. A laser at 632.8 nm was used as a probe beam to measure the power transmitted through the optical setup. The intensity of the transmitted beam could be measured by the Equation (1) [24]:

(1) $${\text{I}}_{\text{t}}={\text{I}}_{\text{o}}{\text{sin}}^{2}\left(\pi \Delta \text{nd}/\lambda \right)$$

Where *λ* was the wavelength of probe beam; d was the thickness of the photochromic film samples; I_o_ was the transmitted intensity of probe beam when the polarizer and analyzer were parallel to each other and the sample was not exposed to the polarized 532 nm laser light.

Figure 11 showed the measured photoinduced birefringence of azo-CPAE copolymers as a function of time. For PAE-allyl20%-azo-20%, at the beginning, no light was transmitted through the analyzer because of the random orientation of azobenzene chromophores. When a linearly polarized 532 nm laser beam (63 mW/cm^2^, at point A) was irradiated on the film of PAE-allyl20%-azo-20%, birefringence signals were induced immediately due to the alignment of azobenzene chromophores perpendicular to the pump laser polarization direction by *trans*-*cis*-*trans* isomerization of azobenzene chromophores. The birefringence exhibited a little decay after the pump laser was switched off (at point B) because of the thermal isomerization from *cis*-form to *trans*-form. The remaining photoinduced anisotropy could be erased with a circularly polarized beam (at point C). PAE-allyl20%-azo40% and PAE-allyl20%-azo60% exhibited photoinduced anisotropy behavior similar to PAE-allyl20%-azo20%, and the relative data are shown in Figure 11. The order of the saturation values of photoinduced birefringence depended on the contents of azobenzene chromophores. PAE-allyl20%, which has no azobenzene, did not exhibit photoinduced anisotropy behavior, as shown in the same figure.

Figure 12 showed the measured photoinduced birefringence of PAE-allyl20%-azo-20%, a typical one of the series, before and after crosslinking (heating at 250 °C for 60 min) as a function of time. The relative data were shown in Table 2. The saturation value of birefringence of PAE-allyl20%-azo-20% before crosslinking lager than that after crosslinking, because the crosslinked net limited the movement of the azobenzene chromophores. But the remnant value of birefringence of the polymer after crosslinking lager than that before crosslinking. The better stability is mainly attributed to the crosslinked net structure, which suppress the relaxation process of the photoinduced alignment.

**Table 2.** Photoinduced birefringence characteristics of PAE-allyl20%-azo20% before and after crosslinking.

Polymer	Saturation value of birefringence	Remnant value of birefringence	Remnant value of birefringence (%)^a^
Before crosslinking	0.0137	0.0111	81
After crosslinking	0.0118	0.0113	96

^a^ Remnant value of birefringence (%) = (remnant value of birefringence)/(saturation value of birefringence) × 100.

Figure 13 showed the photoinduced birefringence of PAE-azo20%, before and after being heated at 250 °C for 60 min as a function of time. To allow qualitative comparison of the birefringence evolution, the same evolution with the value of birefringence normalized to the saturation value of birefringence was shown in Figure 13. It could be seen that PAE-azo20%, which has no crosslinked groups, exhibited no obvious changes in the optical stability before and after being heated at 250 °C for 60 min.

## 4. Conclusions

In this work, we designed and synthesized a series of photoresponsive poly(arylene ether)s with azobenzene chromophores and crosslinked groups by direct copolymerization. The polymers exhibited high glass transition temperature (T_g_ > 147 °C) and good thermal stability (T_d5_ > 365 °C). The influence of thermal crosslinking on the performance of PAE-allyl20%-azo20%, a typical one of the series, was investigated. The T_g_ of PAE-allyl20%-azo20% increased with heating time, indicating the crosslink degree of the polymer increased. After heat-treated for 60 min, T_g_ of the polymer increased to 175 °C from 147 °C before thermal crosslinking. Based on the results of photoinduced birefringence experiment of PAE-allyl20%-azo20%, the polymer possessed remnant value of birefringence after crosslinking larger than that before crosslinking. The results of photoinduced birefringence experiment indicated that crosslinking could suppress the relaxation process of the photoinduced alignment.

## Disclosure statement

No potential conflict of interest was reported by the authors.

## Funding

This work was supported by the Liaoning Province Doctor Startup Fund [number 201501130]; Youth Fund of Liaoning University.
